# Relationship between the timing of preoperative medical visits and day-of-surgery glucose in poorly controlled diabetes

**DOI:** 10.4155/fsoa-2016-0009

**Published:** 2016-06-02

**Authors:** Salma I Patel, Bithika M Thompson, Ryan Y McLemore, M'hamed Temkit, Richard T Schlinkert, Heidi A Apsey, Curtiss B Cook

**Affiliations:** 1 Mayo Clinic, 13400 E Shea Blvd, Scottsdale, AZ 85259, USA; 2 Mayo Clinic Hospital, 5777 E Mayo Blvd, Phoenix, AZ 85054, USA

**Keywords:** anesthesia, diabetes mellitus, hyperglycemia, perioperative, surgery

## Abstract

**Background:**

This study evaluated referral patterns for preoperative evaluations of patients with poorly controlled diabetes mellitus (DM) and determined whether intervals between evaluations and surgery day were associated with preoperative glucose levels.

**Results/methodology:**

In this retrospective analysis of DM patients with a hemoglobin A_1c_ level greater than 8.0%, of the 163 patients who underwent preoperative medical evaluation, only 45% were evaluated by endocrinology. Patients who had surgery earlier than 10 days after the preoperative medical evaluation had preoperative glucose levels 18% higher than those of patients who waited more than 10 days. Preoperative outpatient contact with endocrinology was not associated with preoperative glucose level (p = 0.90).

**Conclusion:**

For poorly controlled DM, more than 10 days are needed to achieve preoperative glycemic control.

## Abstract (lay abstract)

**Lay abstract:** High blood sugar (hyperglycemia) can lead to surgical complications in patients with diabetes mellitus. It is best to identify patients in need of better glucose control before the day of surgery and allow enough time for them to treat their hyperglycemia. This study of patients with poorly controlled diabetes (with hemoglobin A_1c_ levels of more than 8.0%) found that more than 10 days is needed to improve blood sugar control if a high hemoglobin A_1c_ is detected. More effective ways of identifying these patients are needed to permit enough time to treat hyperglycemia before surgery.

**Figure 1.**
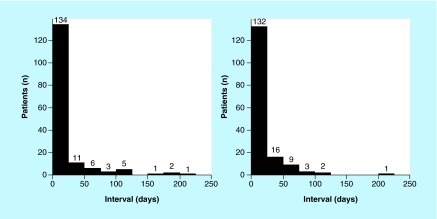
**Distribution of patients according to intervals.** Left: interval between date of preoperative medical evaluation and date of surgery. Right: interval between date of hemoglobin A_1c_ measurement and date of surgery.

**Figure 2.**
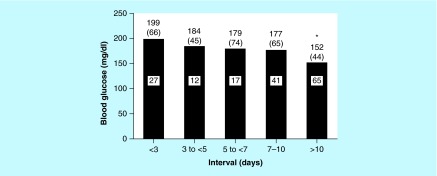
**Preoperative point-of-care blood glucose levels according to intervals.** Intervals are the number of days between the date of the preoperative medical evaluation and the day of surgery. The numbers above the bars indicate mean (SD) blood glucose values, and the numbers within the bars indicate the number of patients in each interval (a glucose value was missing for one patient). The asterisk indicates a significant difference (p < .01) between the mean blood glucose value for the <3-day interval and the >10-day interval.

Diabetes mellitus (DM) is epidemic in the USA, with 9.3% of the population (approximately 29 million people) now having the disease [1]. There are no data on how many patients with DM undergo surgical procedures, but with the rising prevalence of the disease, it is likely that surgical encounters with these patients will also increase. Surgical outcomes are worse among patients who have DM. Morbidity (e.g., surgical site infections) and mortality are increased, especially when perioperative and postoperative glycemic control are poor [2]. Suboptimal glycemic control in DM patients undergoing surgery has been linked to more infections, more reoperations, more frequent organ-related complications (e.g., pneumonia and cardiac dysrhythmias) and a greater risk of death [2]. Consequently, more attention will need to be placed on the quality of care received by patients with DM as they progress through the surgical episode of care.

A history of poor outpatient glycemic control before surgery, which we have previously defined as the preperioperative period for quality improvement purposes, is also associated with poorer surgical outcomes [3]. For instance, elevated hemoglobin A_1c_ (HbA_1c_) levels before surgery are associated with increased surgical site infections, longer inpatient lengths of stay and increased mortality [4–7]. Additionally, hyperglycemia in the immediate preoperative, intraoperative and postoperative periods has been associated with worse patient outcomes [2]. Although the risk of infection has been reported to be less among patients who are undergoing noncardiac surgical procedures and who have lower HbA_1c_ levels [8,9], no prospective studies have actually examined whether optimizing outpatient glycemic control improves surgical outcomes. Nonetheless, given the above retrospective observations that outpatient hyperglycemia is associated with worse outcomes, it would seem reasonable to develop interventions to improve ambulatory glycemic control before the day of surgery, particularly for patients with higher HbA_1c_ levels. In a recent study, interventions for DM patients with HbA_1c_ levels greater than 8.0% decreased preoperative glucose levels [10].

We have been systematically examining the state of diabetes management throughout the spectrum of surgical care at our institution, first studying the inpatient postoperative phase and then the perioperative phase [3,11–14]. However, what has not yet been well investigated are processes of DM care that occur during the period leading up to surgery, and little is known in general about the topic. DM has a high-risk anesthesia classification, and preoperative medical evaluations (POMEs) should be conducted by someone with knowledge of surgical risks. Of specific interest is what minimum amount of time should elapse between a medical evaluation and the day of surgery to see improvements in glycemic control. The goals of this analysis were to determine referral patterns for recommended POMEs and to evaluate whether there is an optimal interval between these evaluations and the surgical date that is associated with lower preoperative blood glucose levels.

## Methods

### Patient selection

This was a retrospective analysis. After this study received institutional review board approval, and as previously described [11–14], we selected for analysis from a surgical database all ambulatory adult patients with DM who underwent an elective surgical procedure under general anesthesia between 1 January 2012 and 30 June 2014. The data set was then restricted to those patients who had an HbA_1c_ greater than 8.0%. This HbA_1c_ cutoff was chosen because patients with this degree of glycemic control are likely at greatest risk for surgical complications. Only single surgical events were included. Per insitutional practice, patients are given a standard set of written instructions regarding dietary restrictions (e.g., the duration of fasting) and medications in preparation for the day of surgery. Data were linked to the laboratory information system to obtain glucose data from the preoperative area. Only point-of-care blood glucose (POC-BG) data were analyzed. POC-BG measurements were performed with an ACCU-CHEK Inform II system (Roche Diagnostics North America). If more than one value was obtained in the preoperative area on the day of surgery, only the maximum value was included in the analysis.

### Data analysis

Data were analyzed to determine whether patients underwent a POME visit and an endocrinology visit. The institution's POME clinic is an outpatient clinic staffed by practitioners with expertise in the assessment and mitigation of risks associated with preplanned anesthesia and surgical procedures. Besides providing an opportunity to evaluate the patient for anesthesia and surgical risks, the POME and endocrinology appointments would provide opportunities to identify the patient with previously unrecognized poorly controlled DM, educate them about the risks associated with hyperglycemia during the perioperative and postoperative phases of care, and potentially introduce interventions to improve glycemic control. To determine the amount of time needed to see an improvement in glycemic control before surgery, the interval between the POME visit and the day of surgery was calculated. The POME visit was chosen as the reference point to calculate the intervals after examination of the referral patterns. The interval between the date of HbA_1c_ measurement and the surgery date was also calculated.

A multivariable linear regression analysis was conducted to determine the relationship between the date of the POME clinic visit and the date of surgery (i.e., the POME–day of surgery interval) and the log-transformed preoperative glucose levels, after adjusting for HbA_1c_, surgical specialty, patient age, sex, race/ethnicity, diabetes duration and contact with the endocrinology service. Patients seen by the endocrinology service were either already established with the endocrinology service or were new referrals made after being identified as having poor glycemic control. Characteristics of patients were compared according to whether they received a preoperative outpatient endocrinology contact. Data are reported as mean (SD) or percentage, as appropriate.

## Results

### Patient characteristics

Between 1 January 2012 and 30 June 2014, a total of 163 patients were identified who had DM and an HbA_1c_ greater than 8.0% and who underwent an elective surgery under general anesthesia (Table 1). Mean age was 63 [12] years, with an average preoperative HbA_1c_ of 8.9% (1%). Most were women, most were white and the mean duration of diabetes was nearly 20 years. The majority of patients were being treated with insulin, either alone or in combination with oral agents. Most procedures involved general surgery, urologic surgery or orthopedic surgery. Nearly all patients were in-state residents. All patients received the recommended POME, but only 45% were seen by the endocrinology service before surgery.

There were some differences between patients that were seen by the endocrinology service and those that were not. Individuals seen by the endocrinology service had higher mean HbA_1c_ levels than those that did not have an endocrinology visit (9.3% [1.2%] vs 8.8% [0.8%]; p < 0.01). The distribution of outpatient DM therapies also varied, with 54% (40 of 74) of the patients with an endocrinology visit receiving insulin monotherapy compared with 31% (28 of 89) of the patients who did not have an endocrinology visit (p = 0.03). Otherwise, there were no differences in age, sex or race/ethnicity (all p > 0.16).

### POME, HbA_1c_ & surgical intervals

Because all patients underwent a POME, the date of the POME visit rather than the date of the endocrinology visit was used as the reference point to calculate the time interval to the surgical date. Therefore, the POME visit was a common access point during which DM and its surgical risks could be discussed with the patient. The mean POME–day of surgery interval was 18 (28) days, but there was marked skewing of the distribution to shorter intervals (median, 8 days) (Figure 1, left panel). The mean interval between the date of HbA_1c_ measurement and the date of surgery was 18 (27) days (Figure 1, right panel). However, the distribution was skewed to shorter intervals (median, 8 days), as it was for the POME–day of surgery interval. For most patients (134 of 163; 82%), HbA_1c_ was measured within 24 h of the POME visit. In 2% of patients (4 of 163), HbA_1c_ was measured more than 24 h after the POME visit and for 15% (25 of 163), HbA_1c_ was measured prior to the POME visit.

### Variables associated with day of surgery glucose level

The mean preoperative maximum POC-BG level was 172 (59) mg/dl. A POC-BG value was not obtained in one patient. Given the range of the POME–day of surgery interval data, intervals were arbitrarily divided into five periods (<3 days, 3 to <5 days, 5 to <7 days, 7–10 days and >10 days), and the mean preoperative POC-BG levels were calculated for each period and compared (Figure 2). The highest mean preoperative POC-BG level was observed in patients when the POME–day of surgery interval was less than 3 days, and the lowest mean POC-BG level was seen when the interval was more than 10 days (p < 0.01 when compared with the <3-day interval). None of the other periods were significantly different when compared with the <3-day interval (all p > 0.13).

With 10 days as the cutoff, Table 2 shows the results of the regression of log-transformed preoperative day ofglucose levels across the variables mentioned above and the importance of timing between the POME and day of surgery. In the adjusted analysis, patients seen in the POME clinic within 10 days before surgery had statistically significant higher mean glucose values (184 [65] mg/dl) in the preoperative area on the date of surgery (p < 0.01) when compared with patients for whom the interval was more than 10 days (152 [44] mg/dl). On average, the group of patients who had surgery 10 days or less after the POME had a glucose level that was 18% higher than that of patients who had surgery more than 10 days after the POME. No other variables (age, HbA_1c_, race/ethnicity, duration of DM, sex, surgery type or outpatient DM therapy) were associated with lower preoperative glucose levels, including whether the endocrinology service had been contacted.

## Discussion

Poor outpatient glycemic control among patients with DM has been associated with increased surgical site infections, longer inpatient lengths of stay and higher mortality [3–7]. Although there are few data on whether optimizing outpatient glycemic control reduces these risks, the development of processes to identify patients who have the worst metabolic control would provide opportunities for intervention. Measures taken to decrease the severity of hyperglycemia before the day of surgery could at least improve the chances of successfully transitioning a patient within a desired glucose range across the continuum of surgical care. Careful planning would also help to avoid disruptions, such as a sudden cancellation on the day of surgery that might occur from unanticipated severe hyperglycemia. The goals of this analysis were to examine referral patterns for medical evaluation of DM patients with an HbA_1c_ greater than 8% and to estimate the ideal window between a preoperative medical review and the day of surgery that might be needed to optimize glucose levels in patients who have poor glucose control.

Our institutional guidelines suggest a referral for an outpatient endocrinology evaluation for patients with an HbA_1c_ level greater than 8.0%. However, endocrinology visits occurred for less than half the patients in this study. This is likely because endocrinology appointments were limited and the short turnaround time between the request for an endocrinology consultation and surgery was inadequate to allow for a visit. Although an endocrinology evaluation for patients with poorly controlled DM was included in the institutional guidelines, this analysis calls into question whether such a referral is needed in order to have a positive effect on preoperative glucose levels. There was no association between involvement of the endocrinology service and the preoperative glucose level on the day of surgery. The prospect of having to postpone an elective surgery because of hyperglycemia may be sufficient motivation for a patient to make dietary changes and adhere more closely to a diabetes medication regimen without the need for specialty care.

Since POME appointments may be more easily available than endocrinology appointments, developing strategies to improve glycemic control before the day of surgery might best be addressed within the POME clinic itself. This approach may have more relevance and impact than an actual endocrinology evaluation. These strategies might include having a dietitian readily available during the visit or even developing a joint effort to position someone from the endocrinology practice (e.g., a diabetes nurse practitioner or physician assistant) directly with the POME practitioners to conduct an evaluation and provide any necessary diabetes education and treatment recommendations. The patient could then be educated on the relationship between hyperglycemia and greater surgical risk and be counseled on the need to improve self-management skills and medication adherence to optimize outcomes. Preoperative intensification of DM therapy may lead to an unintended consequence on the day of surgery in the form of hypoglycemia, and this will have to be tracked as interventions are developed.

Our data show that for most patients HbA_1c_ was measured only within 24 h of the POME visit. If the patients with the poorest metabolic control are going to be identified in a timely manner, knowing the HbA_1c_ sooner would be helpful, preferably through some type of prescreening before the surgical consultation date. This would allow time for appropriate scheduling for other resources that might be needed to address the DM. A recent study [10] reported on an intervention to improve identification of DM patients with HbA_1c_ greater than 8% before surgery and showed a positive effect on glucose levels on the day of surgery. However, the authors of that study also struggled with very short intervals between the identification of the patient and the surgical date and admitted that implementing a case finding and intervention strategy similar to theirs so close to surgery would be resource and manpower intensive. The present study was intended to examine the current state of our institution's practice, rather than to conduct an intervention, and to ask whether there might be an optimal time between identification of similar cases of poor glucose control and the date of surgery. A common barrier to improving preoperative glucose levels that many centers may face, as shown by the present study and others, is the narrow window for identifying high-risk patients and implementing interventions [10].

Our data showed that, at least for these patients with the highest HbA_1c_, at least 10 days would be needed prior to the day of surgery to achieve improvements in glucose control. A recent study [15] found that surgical complications were higher even in patients with far better outpatient glycemic control than the ones analyzed here. To improve the care of a patient with DM, the process of care should be altered to allow enough time to introduce changes in management that would decrease the severity of hyperglycemia for all patients with DM prior to surgery. However, it is unlikely that patients will want to wait the several months needed to see improvements in HbA_1c_ before being cleared for surgery. Therefore, glucose, which can reflect changes more quickly than HbA_1c_, may be the better target of interventions designed to improve preoperative glycemic control.

The retrospective nature of the present study limits the ability to determine specifically what interventions were implemented at the POME or the endocrinology practice visits that might have led to better glycemic control on the day of surgery. It is possible that patients simply adhered to lifestyle or medication recommendations after they were counseled about the need to improve glycemic control. Alternatively, existing medical therapies may have been intensified. A systematic management approach should be developed that includes algorithms for rapid induction or intensification of pharmacologic therapy when indicated. Additionally, the current sample size was too small to allow stratification of analyses based on surgery type. There has been recent controversy over the use of POC-BG technology in the hospital, particularly in critical care or operative settings, but alternative technologies for accurate and rapid turnaround glucose measurements are limited [16]. Finally, the findings are from a single institution. Nonetheless, despite its limitations, this study did yield information supporting the hypothesis that in the days leading up to surgery, a minimum amount of time should be allowed to improve the patient's glycemic control for the day of surgery.

## Future perspective

Processes of DM care leading up to surgery have not been studied sufficiently. This analysis selected those individuals with the highest HbA_1c_ levels. For patients with better outpatient glycemic control, a shorter interval between a medical evaluation and surgery may be sufficient to adjust the patient's glucose level before surgery and should be assessed in future analyses. Collaborative efforts between the various practitioners involved with the surgical process and patients should be encouraged and tested to yield the best glycemic outcomes. Finally, additional studies are required to determine the optimal amount of time and the safest and most effective ways of treating hyperglycemia in the days leading up to surgery.

**Table 1.** **Demographics of 163 patients who had diabetes mellitus and underwent an elective surgical procedure.**

**Variable**	**Result**
Age, years	63 (12)
Female	85 (52)
White race	128 (79)
Preoperative HbA_1c_ (%)	8.9 (1.0)
Duration of DM, years^†^	18 (13)
Outpatient DM therapy	
– Diet	9 (5)
– Oral agents and insulin	35 (22)
– Oral agents only	51 (31)
– Insulin monotherapy	68 (42)
Type of surgery	
– General	38 (23)
– Urologic	36 (22)
– Orthopedic	32 (20)
– Otolaryngologic	20 (12)
– Gynecologic	16 (10)
– Plastic	14 (9)
– Neurologic	4 (2)
– Cardiothoracic	3 (2)
In-state residence	148 (91)
Underwent a POME	163 (100)
POME–surgery interval, days	18 (28)
Endocrinology evaluation	74 (45)

^†^Continuous data are expressed as mean (SD); categorical data as number of patients (percentage of sample).

Available for 142 patients.

DM: Diabetes mellitus; HbA_1c_: Hemoglobin A_1c_; POME: Preoperative medical evaluation.

**Table 2.** **Variables associated with preoperative glucose levels.**

**Variable**	**Estimated change (%)**	**95% CI**	**p-value**
**Age (years)**	0.04	-0.49 to 0.57	0.88
**HbA_1c_ (%)**	0.36	-5.36 to 6.42	0.90
**White race (vs nonwhite)**	-4.47	-17.35 to 10.41	0.53
**Duration of DM (years)**	-0.17	-0.68 to 0.35	0.51
**Female**	-0.07	-11.56 to 12.92	0.99
**Type of surgery**^†^	9.50	-4.47 to 25.50	0.19
**Endocrinology care**	0.79	-10.59 to 13.63	0.90
**Outpatient DM therapy^‡^**	-3.5	-10.1 to 3.5	0.32
Diet	-1.61	-16.28 to 15.64	0.84
Oral agents and insulin	-3.59	-17.86 to 13.16	0.65
Oral agents only	-23.88	-42.22 to .29	0.05
**POME-surgery date interval ≤10 days^§^**	18.13	4.51 to 33.53	<0.01

^‡,†,§^Referent general surgery.

Referent insulin therapy.

Versus >10 days.

DM: Diabetes mellitus; HbA1c: Hemoglobin A1c; POME: Preoperative medical evaluation.

**Executive summary**

Poor outpatient glycemic control is associated with worse surgical outcomes among patients with diabetes mellitus (DM).
Optimizing glucose levels before surgery can potentially minimize complications.
The goals of this analysis were:
  To determine referral patterns for recommended preoperative medical evaluations (POMEs).
  To evaluate whether there is an optimal interval between POMEs and the surgical date that is associated with lower preoperative blood glucose levels.

**Methods**

Retrospective analysis of patients with DM undergoing elective surgery.
Limited to individuals with hemoglobin A_1c_ levels greater than 8.0%.
Interval between a POME and the surgery date was calculated.
Relationship between the interval and the glucose level on the day of surgery was evaluated.

**Results**

A total of 163 patients underwent POMEs.
Only 45% were seen by the endocrinology service preoperatively.
Patients who had surgery within 10 days after the POME had preoperative glucose levels 18% higher than those of patients who waited more than 10 days.
Outpatient contact with the endocrinology service before surgery was not associated with preoperative glucose level.

**Conclusion**

For patients with poorly controlled DM, more than 10 days are needed to achieve preoperative glycemic control.
Additional studies are required to determine the safest and most effective ways of treating hyperglycemia in the days leading up to surgery.

## References

[1] Centers for Disease Control and Prevention National diabetes statistics report, 2014: estimates of diabetes and its burden in the United States. http://www.cdc.gov/diabetes/pubs/statsreport14/national-diabetes-report-web.pdf.

[2] Coan KE, Apsey HA, Schlinkert RT, Stearns JD, Cook CB (2014). Managing diabetes mellitus in the surgical patient. *Diabetes Manag.*.

[3] Shah M, Apsey HA, Stearns JD, Schlinkert RT, Seifert KM, Cook CB (2014). Guidelines to improve perioperative management of diabetes mellitus: an example of a successful quality initiative. *Diabetes Manag.*.

[4] Feringa HH, Vidakovic R, Karagiannis SE (2008). Impaired glucose regulation, elevated glycated haemoglobin and cardiac ischaemic events in vascular surgery patients. *Diabet. Med.*.

[5] Halkos ME, Lattouf OM, Puskas JD (2008). Elevated preoperative hemoglobin A1c level is associated with reduced long-term survival after coronary artery bypass surgery. *Ann. Thorac. Surg.*.

[6] Halkos ME, Puskas JD, Lattouf OM, Kilgo P, Kerendi F, Song HK (2008). Elevated preoperative hemoglobin A1c level is predictive of adverse events after coronary artery bypass surgery. *J. Thorac. Cardiovasc. Surg.*.

[7] Sato H, Carvalho G, Sato T, Lattermann R, Matsukawa T, Schricker T (2010). The association of preoperative glycemic control, intraoperative insulin sensitivity, and outcomes after cardiac surgery. *J. Clin. Endocrinol. Metab.*.

[8] Dronge AS, Perkal MF, Kancir S, Concato J, Aslan M, Rosenthal RA (2006). Long-term glycemic control and postoperative infectious complications. *Arch. Surg.*.

[9] Underwood P, Askari R, Hurwitz S, Chamarthi B, Garg R (2014). Preoperative A1C and clinical outcomes in patients with diabetes undergoing major noncardiac surgical procedures. *Diabetes Care*.

[10] Underwood P, Seiden J, Carbone K (2015). Early identification of individuals with poorly controlled diabetes undergoing elective surgery: improving A1C testing in the preoperative period. *Endocr. Pract.*.

[11] Coan KE, Schlinkert AB, Beck BR (2013). Clinical inertia during postoperative management of diabetes mellitus: relationship between hyperglycemia and insulin therapy intensification. *J. Diabetes Sci. Technol.*.

[12] Apsey HA, Coan KE, Castro JC, Jameson KA, Schlinkert RT, Cook CB (2014). Overcoming clinical inertia in the management of postoperative patients with diabetes. *Endocr. Pract.*.

[13] Coan KE, Schlinkert AB, Beck BR (2013). Perioperative management of patients with diabetes undergoing ambulatory elective surgery. *J. Diabetes Sci. Technol.*.

[14] Udovcic M, Castro JC, Apsey HA, Stearns JD, Schlinkert RT, Cook CB (2015). Guidelines to improve perioperative management of diabetes mellitus: assessment of the impact of change across time. *Endocr. Pract.*.

[15] Goodenough CJ, Liang MK, Nguyen MT (2015). Preoperative glycosylated hemoglobin and postoperative glucose together predict major complications after abdominal surgery. *J. Am. Coll. Surg.*.

[16] Klonoff DC, Draznin B, Drincic A (2015). PRIDE statement on the need for a moratorium on the CMS plan to cite hospitals for performing point-of-care capillary blood glucose monitoring on critically ill patients. *J. Clin. Endocrinol. Metab.*.

